# A novel protective role for microRNA-3135b in Golgi apparatus fragmentation induced by chemotherapy via GOLPH3/AKT1/mTOR axis in colorectal cancer cells

**DOI:** 10.1038/s41598-020-67550-0

**Published:** 2020-06-29

**Authors:** Stephanie I. Núñez-Olvera, Bibiana Chávez-Munguía, María Cruz del Rocío Terrones-Gurrola, Laurence A. Marchat, Jonathan Puente-Rivera, Erika Ruíz-García, Alma D. Campos-Parra, Carlos Vázquez-Calzada, Erik R. Lizárraga-Verdugo, Rosalío Ramos-Payán, Yarely M. Salinas-Vera, César López-Camarillo

**Affiliations:** 1 Posgrado en Ciencias Genómicas, Universidad Autónoma de la Ciudad de México, San Lorenzo 290 col del Valle, C.P. 03100 Mexico City, México; 2 Departamento de Infectómica y Patogénesis Molecular, CINVESTAV-IPN, Mexico City, México; 3 Coordinación Académica Región Altiplano, Universidad Autónoma de San Luis Potosí, San Luis Potosí, México; 4 Programa en Biomedicina Molecular y Red de Biotecnología, Instituto Politécnico Nacional, Mexico City, México; 5 Departamento de Ecología Funcional, Instituto de Ecología, Universidad Nacional Autónoma de México, Mexico City, México; 6 Laboratorio de Medicina Translacional y Departamento de Tumores Gastro-Intestinales, Instituto Nacional de Cancerología, Mexico City, México; 7 Laboratorio de Genómica, Instituto Nacional de Cancerología, Mexico City, México; 8 Facultad de Ciencias Químicas Biológicas, Universidad Autónoma de Sinaloa, Sinaloa, México

**Keywords:** Cancer, Oncology

## Abstract

Chemotherapy activates a novel cytoplasmic DNA damage response resulting in Golgi apparatus fragmentation and cancer cell survival. This mechanism is regulated by Golgi phosphoprotein-3 (GOLPH3)/Myo18A/F-actin axis. Analyzing the functions of miR-3135b, a small non-coding RNA with unknown functions, we found that its forced overexpression attenuates the Golgi apparatus fragmentation induced by chemotherapeutic drugs in colorectal cancer (CRC) cells. First, we found that miR-3135b is downregulated in CRC cell lines and clinical tumors. Bioinformatic predictions showed that miR-3135b could be regulating protein-encoding genes involved in cell survival, resistance to chemotherapy, and Golgi dynamics. In agreement, ectopic transfection of miR-3135b in HCT-15 cancer cells significantly inhibited cell proliferation, sensitized cells to 5-fluoruracil (5-FU), and promoted late apoptosis and necrosis. Also, miR-3135b overexpression impaired the cell cycle progression in HCT-15 and SW-480 cancer cells. Because *GOLPH3*, a gene involved in maintenance of Golgi structure, was predicted as a potential target of miR-3135b, we studied their functional relationships in response to DNA damage induced by chemotherapy. Immunofluorescence and cellular ultrastructure experiments using antibodies against TGN38 protein, a trans-Golgi network marker, showed that 5-FU and doxorubicin treatments result in an apoptosis-independent stacks dispersal of the Golgi ribbon structure in both HCT-15 and SW-480 cells. Remarkably, these cellular effects were dramatically hindered by transfection of miR-3135b mimics. In addition, our functional studies confirmed that miR-3135b binds to the 3′-UTR of GOLPH3 proto-oncogene, and also reduces the levels of p-AKT1 (Ser473) and p-mTOR (Ser2448) signaling transducers, which are key in cell survival and autophagy activation. Moreover, we found that after treatment with 5-FU, TGN38 factor coimmunolocalizes with beclin-1 autophagic protein in discrete structures associated with the fragmented Golgi, suggesting that the activation of pro-survival autophagy is linked to loss of Golgi integrity. These cellular effects in autophagy and Golgi dispersal were reversed by miR-3135b. In summary, we provided experimental evidence suggesting for the first time a novel role for miR-3135b in the protection of chemotherapy-induced Golgi fragmentation via GOLPH3/AKT1/mTOR axis and protective autophagy in colorectal cancer cells.

## Introduction

Colorectal cancer (CRC) is the second leading cause of cancer death worldwide^1^. Unfortunately, more than 80% of patients are diagnosed in advanced stages of disease, which results in low response to therapy and poor survival^2^. Despite the different treatment strategies which include sequential combinations of radiotherapy and chemotherapy with 5-fluouracil (5-FU), oxaliplatin, and capecitabine^3^, the rate of clinical response of CRC patients remains low due to increased cellular resistance to therapy^4^. This adverse situation urged to define novel therapeutic approaches to overcome resistance and ameliorate the patient clinical response to actual therapies. Over the past decades, diverse molecular mechanisms for therapy resistance mainly operating by the activation of the so-called “nuclear DNA-damage response” have been described in human cancers. Nevertheless, it was recently reported in an outstanding study that, in response to chemotherapeutic treatments with DNA-damaging agents, a novel cytoplasmic DNA damage response is triggered, resulting in Golgi apparatus dispersal and cancer cell survival^5,6^. Farber-Katz and coworkers^5^ demonstrated that the nuclear kinase DNA-PK is activated, which in turn phosphorylates the Golgi phosphoprotein-3 (GOLPH3) leading to strong interactions of GOLPH3 with unconventional myosin XVIIIa (MYO18a) that consequently increases the tensile forces with F-actin cytoskeleton resulting in Golgi apparatus fragmentation and dispersal. Notably, this aberrant Golgi-dispersion was necessary to support cell survival and conferred resistance to DNA-damaging agents^7^. Golgi-dispersion has also been associated with changes in glycosylation levels characterized by increased sialylation, protection from apoptosis and suggested to confer resistance to therapy^8,9^. Indeed, the interactions between GOLPH3 and the sialyltransferase ST6GAL1 modulate the sialylation of oncogenic receptor tyrosine kinases promoting the increase of AKT1-mTOR signaling transduction. In addition, recent experimental findings suggested that Golgi membranes are essential sites for the assemblage and production of autophagic-vesicles that nucleate the membranous structures shaping the phagophore formation during the early stages of autophagy^10^.

In this scenario, GOLPH3 functions are relevant for the activation of the cytoplasmic DNA damage response and Golgi apparatus integrity. GOLPH3 is a trans-Golgi network membrane protein highly conserved from yeast to human that plays crucial roles in Golgi dynamics and vesicular trafficking. Notably, GOLPH3 oncogene amplification at chromosome 5p13 and protein overexpression have been documented in a high proportion of human malignancies, including colorectal cancer, and frequently associated with a poor prognosis, and chemotherapy resistance, which makes it an attractive therapeutic target and raises interesting questions about how GOLPH3 and the Golgi structure integrity contribute to cancer^5,11–18^. However, whether non-coding small RNAs with potential tumor suppressor functions participate in the aforementioned cytoplasmic DNA damage response by regulating the expression of GOLPH3 and related players, including AKT1-mTOR signaling transducers, is largely unknown. MicroRNAs are small non-coding RNAs of 25 nucleotides length which are estimated to modulate over 30% of protein-encoding genes by translational repression or degradation of transcripts, thus playing essential roles in diverse human diseases including cancer^19–23^. In the present investigation, we have studied the protective functions of miR-3135b in Golgi structure integrity and pro-survival autophagy activation in response to 5-FU and doxorubicin drugs. Our data suggest that miR-3135b is a novel tumor suppressor with a potential role in the regulation of the GOLPH3/AKT1/mTOR axis to maintain Golgi integrity, which adds a piece in the puzzle of the novel cytoplasmic DNA damage response to DNA-damaging agents in CRC cells.

## Results

### MicroRNA-3135b expression is repressed in colorectal cancer cells and tumors

**Figure 1:**
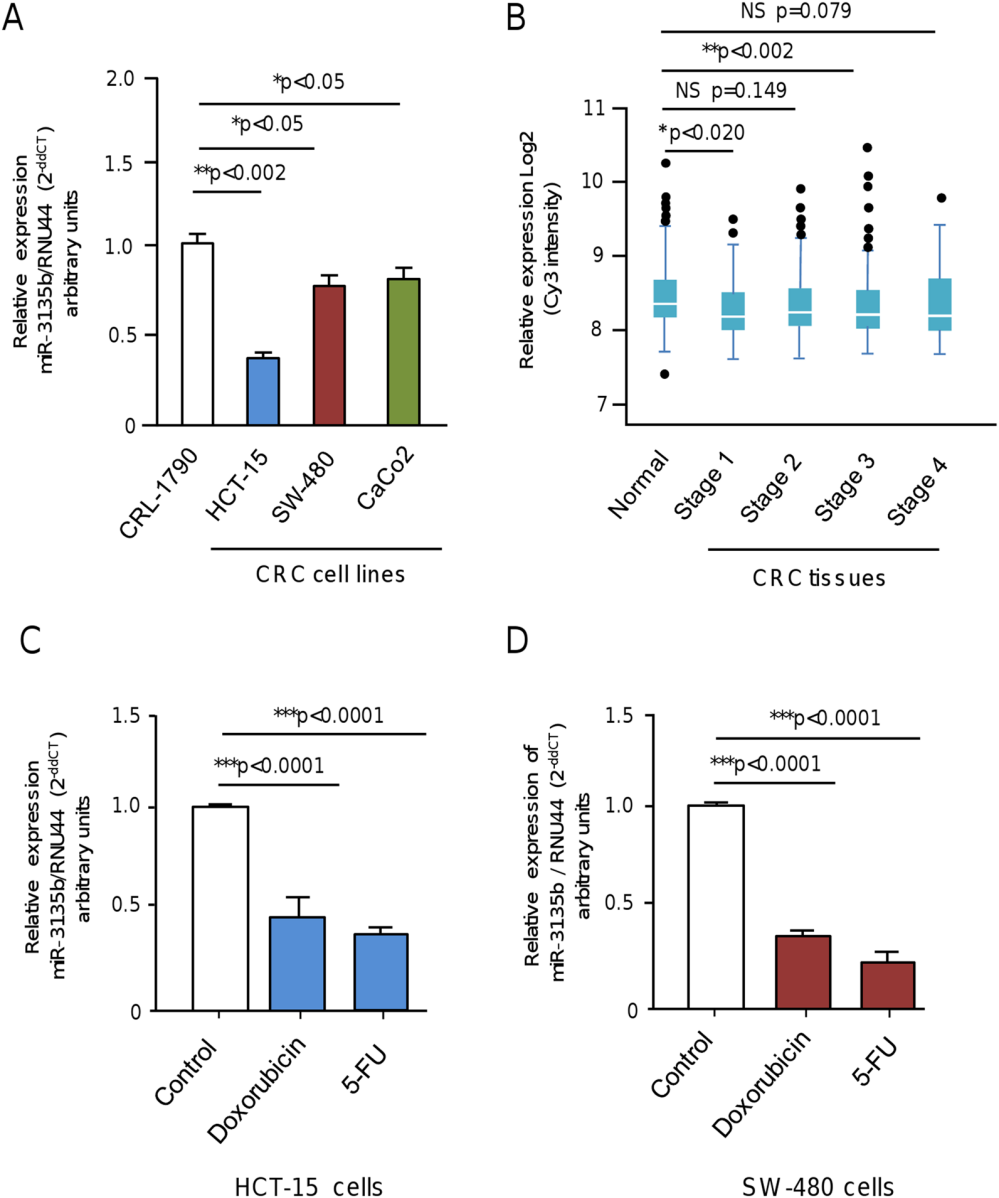
Relative expression of miR-3135b in colorectal cancer cell lines and tumors. (**A**) qRT-PCR assays for miR-3135b expression in colorectal cancer cell lines. The bars indicate the relative expression of miR-3135b in the HCT-15, SW-480, CaCo2 colorectal cancer (CRC) cells and normal CRL-1790 colon cells. Data were normalized with the endogenous small-nucleolar RNU44. Bars represent the mean of three independent experiments ± S.D. Significance was assessed using analysis of variance ANOVA followed by Tukey post-hoc analysis with a value of *p* < 0.05 as significant. (**B**) Box plots showing the relative expression log2 of miR-3135b in normal colon tissues and colon adenocarcinoma stages 1–4. Data were obtained from 1893 normal/carcinoma-paired samples and 290 adenoma tissue samples from a previous study (24) deposited in NCBI GEO database (GSE115513). Expression analysis was carried out using the Limma package in R (https://www.bioconductor.org/packages/release/bioc/html/limma.html) which employs gene-wise linear models and empirical Bayes method. Multiple testing correction by considering an FDR < 0.05 as statistically significant was used. Horizontal lines in the middle of box plot denote the median. Lines extending from the boxes indicate variability outside the upper and lower quartiles. NS, Non-significant. (**C**–**D**) qRT-PCR assays for miR-3135b expression in (**C**) HCT-15 and (**D**) SW-480 colorectal cancer cell lines treated with doxorubicin and 5-FU compared to baseline levels. Data were normalized with the endogenous small-nucleolar RNU44. Statistical significance was determined by Student's t-test and *p* < 0.05 were considered statistically significant.

To initiate the study of miR-3135b, a non-coding RNA located in chromosome 6p21.32 with no previous involvement in human cancers, we analyzed its expression levels in three CRC cell lines and normal colon cells using stem loop qRT-PCR assays. Data showed that miR-3135b levels are significantly downregulated in HCT-15, SW-480 and Caco2 cancer cells in comparison with normal CRL-1790 colon cells (Fig. 1A). To further investigate the clinical relevance of miR-3135b, we also analyzed its expression during the early and late stages of carcinogenesis using previous published data obtained from a miRNAs profiling of 1893 normal/colorectal carcinoma-paired samples, and 290 adenoma tissues samples deposited in NCBI GEO database (GSE115513)^24^. Results showed a slight but significant downregulation of miR-3135b expression in CRC stages 1 and 3 of tumor progression relative to normal colon tissues, whereas no significant changes in miR-3135b levels were detected between normal tissues and CRC stages 2 and 4 (Fig. 1B). Also, we evaluated if miR-3135b expression could be regulated by chemotherapeutic drugs. Data evidenced that the low miR-3135b expression is even more suppressed after DNA damage induced by 5-FU and doxorubicin treatments in comparison to untreated controls in both HCT-15 and SW-480 cancer cells (Fig. 1C,D).

### MicroRNA 3135b is predicted to regulate genes involved in Golgi apparatus integrity

**Figure 2:**
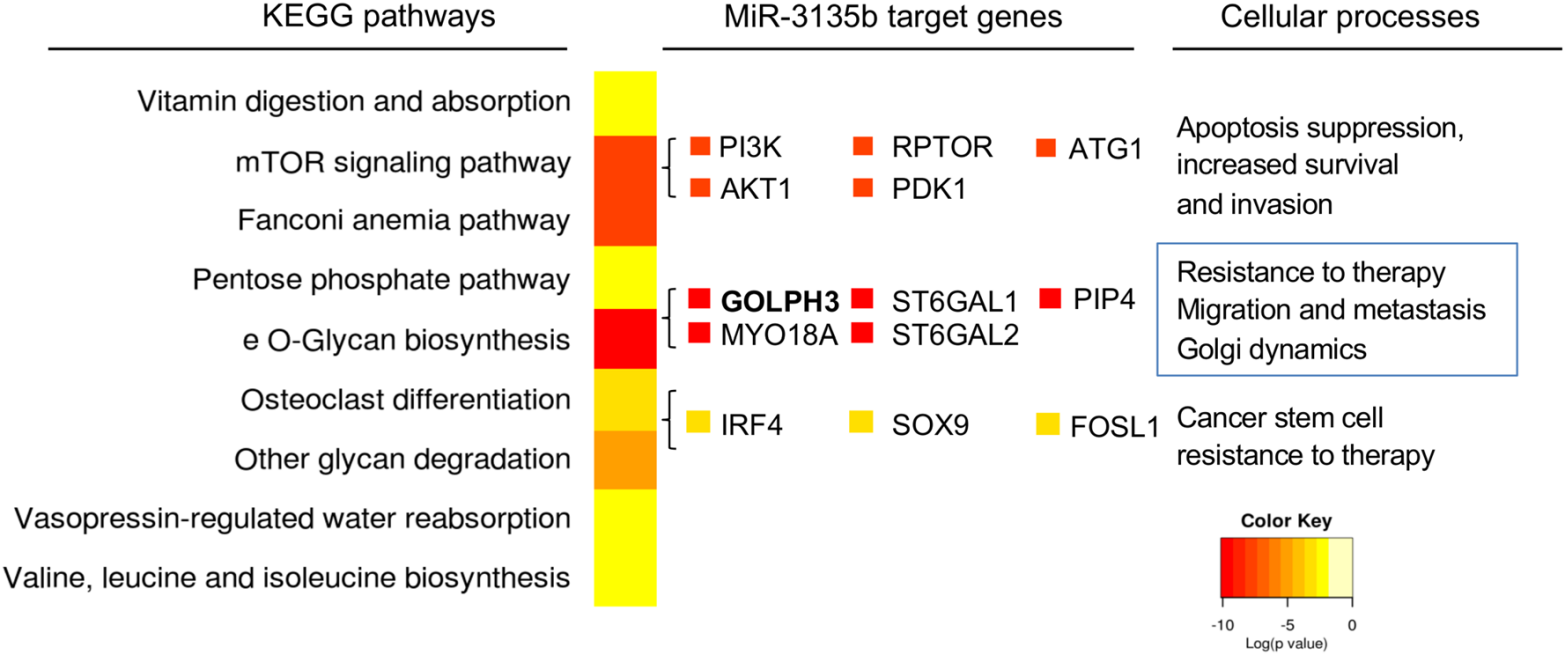
Supervised hierarchical clustering of signaling pathways and process affected by miR-3135b. The image shows the most significant cellular processes and signaling pathways modulated by miR-3135b according to microT-CDS software. The colors indicate the *p* value of the prediction, *p* < 0.05 was considered statistically significant as described by DIANA-miRPath Software v3, 2018.

To investigate the functions of miR-3135b, we searched for its potential gene targets and associated signaling pathways by performing a bioinformatic analysis using TargetScan and miRmap softwares. Results revealed that a number of genes involved in Golgi structure and functions, such as *GOLPH3, MYO18A, SET1, ST6GAL1* and *ST6GAL2*, contain potential miR-3135b binding sites at their 3′-UTRs, suggesting that they could be regulated at the posttranscriptional level (Fig. 2). Moreover, several protein-encoding genes involved in apoptosis, cell survival, stemness and resistance to chemotherapy, as well as in AKT1-mTOR signaling pathways were found as potentially modulated by miR-3135b (Fig. 2, Table 1).

**Table 1:** Prediction of genes potentially regulated by miR-3135b in the AKT-mTOR pathways according to miRmap (https://mirmap.ezlab.org/) and targetscan (https://www.targetscan.org/vert_72/) softwares.

microRNA	Signaling pathway	Target gene	miRmap score	Targetscan score
miR-3135b	AKT-mTOR	AKT1	99.06	74.0
	AKT-mTOR	PIK3CA	95.09	78.0
	AKT-mTOR	PRAS40	90.68	81.0
	AKT-mTOR	S6K	70.35	56.0
	AKT-mTOR	ATG1	39.17	82.0
	AKT-mTOR	PDK1	No determined	32.0
	AKT-mTOR	RAPTOR	4.51	55.0

### MicroRNA-3135b impairs proliferation, cell cycle progression and promotes late apoptosis and necrosis

**Figure 3:**
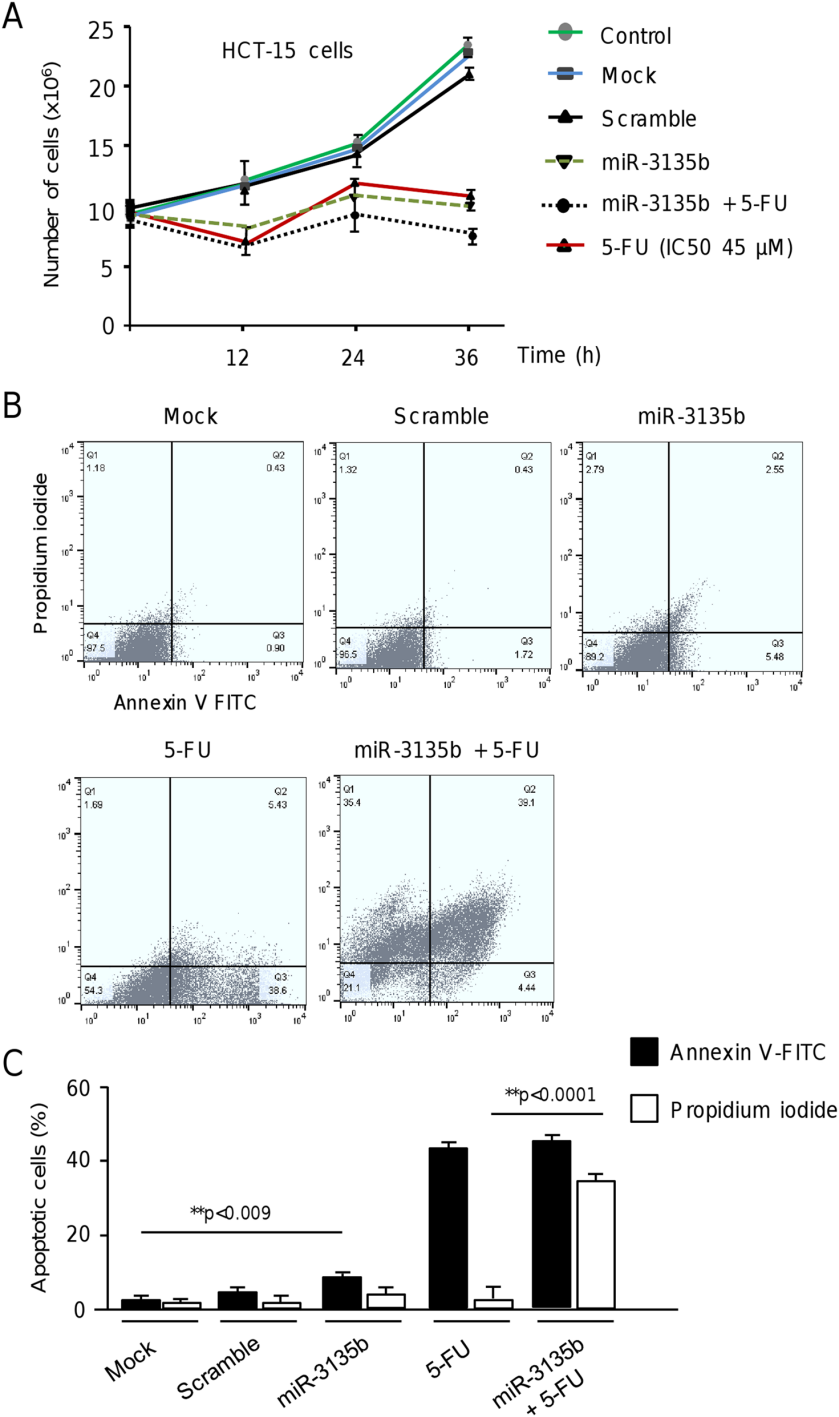
Cell proliferation and apoptosis assays. (**A**) MTT assays. HCT-15 cells were transfected with scramble or miR-3135b and exposed to 5-FU therapy; subsequently cell viability was assessed at 0, 12, 24 and 36 h. (**B**) Apoptosis was measured by flow cytometric analysis by double staining with annexin V-FITC and propidium iodide (PI). The plots represent apoptotic (annexin-V positive and PI-negative) and necrotic (PI-positive) events. (**C**) Graphical representation of the proportion of apoptotic cells according to annexin-V or PI staining in B. The error bars represent the standard error of the mean of three experiments in triplicate and *p* values < 0.05 were considered statistically significant.

**Figure 4:**
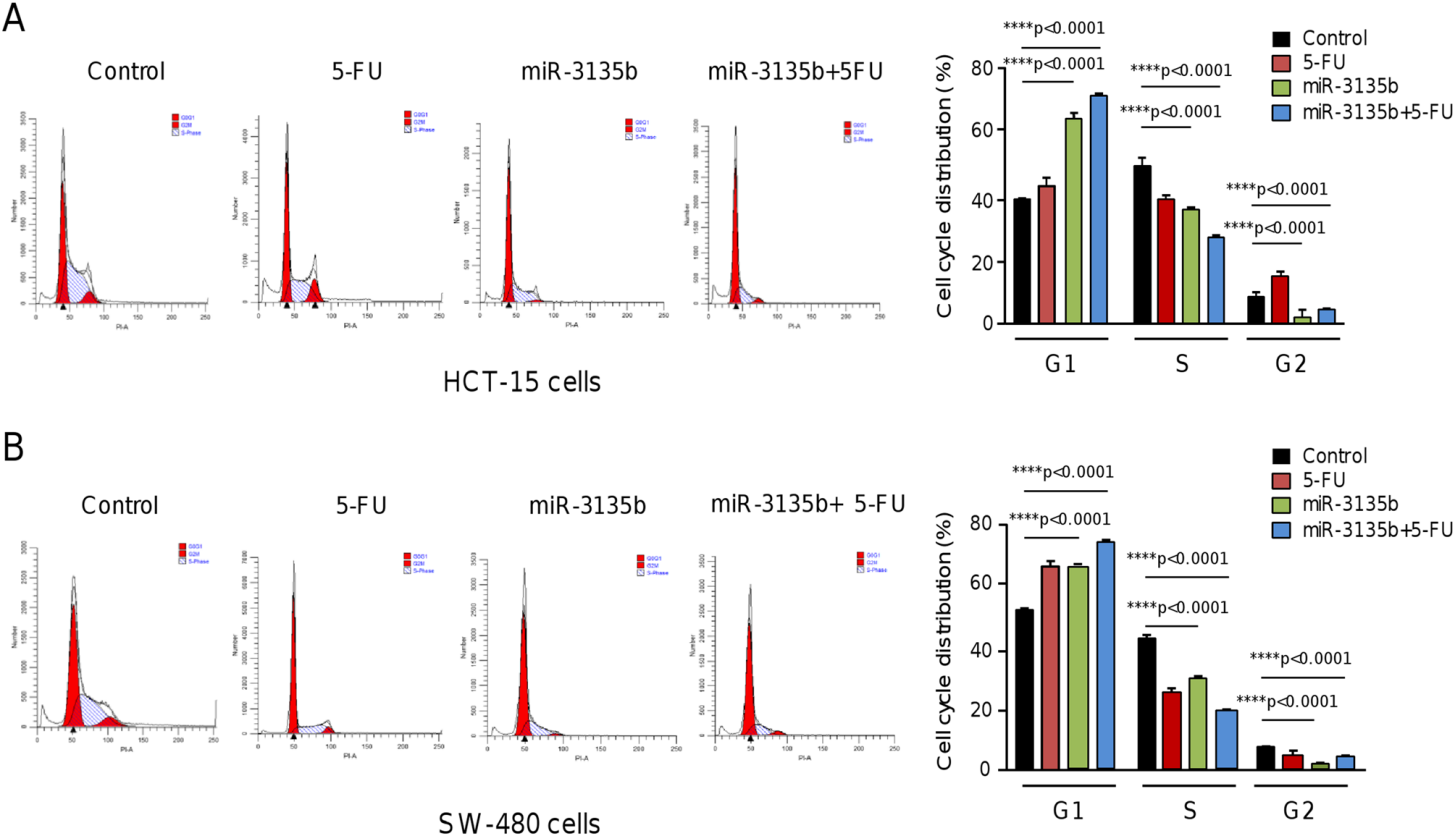
Effects of miR-3135b on cell cycle progression. (**A**) HCT-15 and (**B**) SW-480 cancer cells were transfected with miR-3135b and treated or not with 5-FU. After 48 h non treated control and miR-3135b treated cells were collected and the percentages of cells in the different cell cycle phases were quantified by flow cytometry. *p* values < 0.05 were considered to be statistically significant.

Next, we wondered if miR-3135b could regulate cell proliferation, and apoptosis, and sensitizes CRC cells to 5-FU therapy. Results showed that proliferation of HCT-15 cells transfected with miR-3135b mimic is significantly decreased in comparison with non-transfected and scramble transfected control cells at 12, 24 and 36 h (Fig. 3A). Next, we calculated the half-maximal inhibitory concentrations (IC_50_) for 5-FU in HCT-15 cells using MTT assays, and performed combined treatments using miR-3135b mimic and 5-FU. Data evidenced that restoration of miR-3135b slightly but significantly sensitizes HCT-15 cells to 5-FU treatment in comparison with 5-FU monotherapy after 24 h and 36 h (Fig. 3A). To evaluate the effects of miR-3135b on early and late apoptosis, we performed double staining of cells with annexin V-FITC (AN) and propidium iodide (PI). The proportion of cells that exhibit AN−/PI−, AN+/PI−, AN−/PI+ and AN+/PI+ staining representing normal, early apoptotic, necrotic and late apoptotic cell populations, respectively, was determined using fluorescence activated cell sorting. Data showed a significant increase in the percentage of apoptotic cells after intervention with miR-3135b mimic in comparison to mock and scramble transfected controls (Fig. 3B,C). Moreover, a dramatic increment of cells in late apoptosis and necrosis after miR-3135b plus 5-FU combined treatments was found (Fig. 3B, C). Next, we evaluated the effects miR-3135b and combined treatments in cell cycle progression of CRC cell lines. Data indicated that transfection of miR-3135b, alone or in presence of 5-FU results in a significant increase of the percentage of cells in G1 phase and a diminution of cells in S1 phase relative to untreated control, suggesting that inhibition of cell proliferation could be due, at least in part, to cell cycle arrest (Fig. 4A,B). This effect was more pronounced in HCT-15 cell line in comparison to SW-480 cancer cells.

### MicroRNA-3135b attenuates Golgi disruption in response to therapy

**Figure 5:**
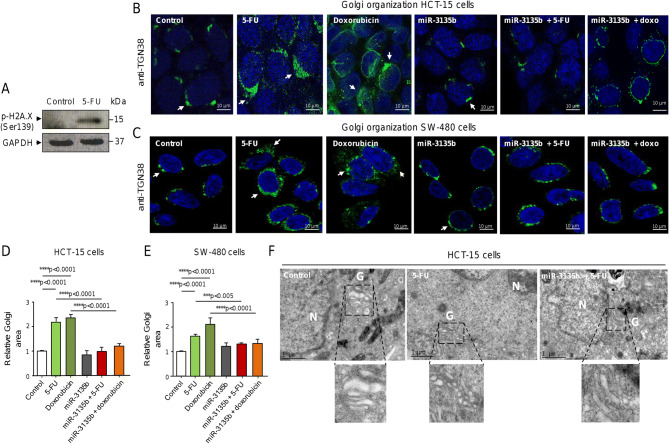
Drugs-induced Golgi apparatus dispersal is attenuated by miR-3135b in HCT-15 and SW-480 cells. (**A**) Detection of DNA damage after 5-FU treatment. Cropped images from immunoblots using antibodies against phosphorylated-histone H2A.X (Ser139) after incubation with 5-FU for 12 h. GADPH was used as endogenous loading control. Complete developed Western blot membranes are provided in Supplementary data 1. Immunofluorescence assays in (**B**) HCT-15 and (**C**) SW-480 cells using antibodies against TGN38 trans-Golgi network protein (green channel) treated with drugs and/or miR-3135b as indicates. DAPI staining was used to visualize nuclei (blue channel). (**D**–**E**) Quantification of relative Golgi area based on TGN38 fluorescent signal intensity found between treatments in B and C panels. (**F**) Transmission electronic microscopy (TEM) micrographs of control, 5-FU, and miR-3135b plus 5-FU treated cells showing the morphological changes in Golgi ultrastructure. Bottom images show a zoom of the Golgi. N = nuclei. G = Golgi. The error bars represent the standard error of the mean of three experiments in triplicate and *p* values < 0.05 were considered statistically significant.

The induction of DNA double strand breaks (DSBs) by chemotherapeutic drugs has been shown to activate a cytoplasmic DNA damage response, i.e. the GOLPH3/MYO18A/F-actin pathway, to promote Golgi apparatus dispersal and cancer cells survival^5^. Our bioinformatics analyses identified the GOLPH3 gene as a potential target of miR-3135b (Fig. 2). GOLPH3 is a proto-oncogene involved in the maintenance of Golgi architecture and vesicular trafficking system, as well as in the activation of AKT and mTOR transducers involved in eukaryotic cells survival. Therefore, we decided to evaluate the potential role of miR-3135b in the regulation of GOLPH3 and Golgi apparatus structure in response to chemotherapy treatments. First, we examined the levels of phosphorylated H2A.X (Ser139) histone, an early DNA damage marker, in HCT-15 cells exposed to 5-FU. As expected, immunoblotting results confirmed a significant increase in phospho-H2A.X (Ser139) levels after 5-FU intervention in comparison to non-treated cells, indicative of the presence of DNA DSBs and DNA damage (Fig. 5A). Next, we established the model of Golgi fragmentation using treatments with 5-FU and doxorubicin compounds and tracked the morphologic alterations in the organelle structure using antibodies against TGN38, a trans-Golgi network protein marker, followed by immunofluorescence and confocal microscopy assays in CRC cells. Data showed that TGN38 signal is located at the trans-Golgi network in ring-shaped structures around the nucleus in control HCT-15 cells (Fig. 5B). Quantitative analysis of TGN38-immunostained cells indicated that both 5-FU and doxorubicin drugs significantly alter the Golgi apparatus structure inducing the stacks dispersal of the perinuclear ribbon (Fig. 5B, middle panels). These morphologic changes were accompanied by a significant increase in the relative Golgi area of HCT-15 cells treated with 5-FU and doxorubicin relative to non-treated control (Fig. 5B,D). Interestingly, the transfection of miR-3135b mimics in combination with the drugs dramatically hindered the Golgi fragmentation induced by 5-FU or doxorubicin alone and significantly reduced the Golgi area close to untreated control (Fig. 5B, D). These findings were confirmed in a second CRC cell line, SW-480. Likewise, the TGN38 signal was found at the trans-Golgi network surrounding the nucleus in SW-480 cells (Fig. 5C), in a very similar stain pattern as found in HCT-15 cells (Fig. 5B). The fragmentation of the Golgi structure induced by 5-FU and doxorubicin was also significantly impaired after transfection of miR-3135b mimic in comparison with non-transfected control in SW-480 cells (Fig. 5C,E). These data demonstrate that drug treatments induced significant alterations of the Golgi ribbon ultrastructure depicted as stacks dispersal, and highlight the protective effect of miR-3135b on Golgi integrity in two different CRC cell lines. To further confirm the immunofluorescence findings, a cellular ultrastructure analysis of Golgi apparatus using transmission electronic microscopy (TEM) was performed in the same conditions as previously described. TEM results confirmed the presence of significant changes in the organelle ultrastructure associated with stacks dispersal of the Golgi ribbon structure after 5-FU treatments, and corroborated the protective role of miR-3135b on Golgi integrity in HCT-15 cells (Fig. 5F).

### Golgi dispersal occurs independently of apoptosis in CRC cells

**Figure 6:**
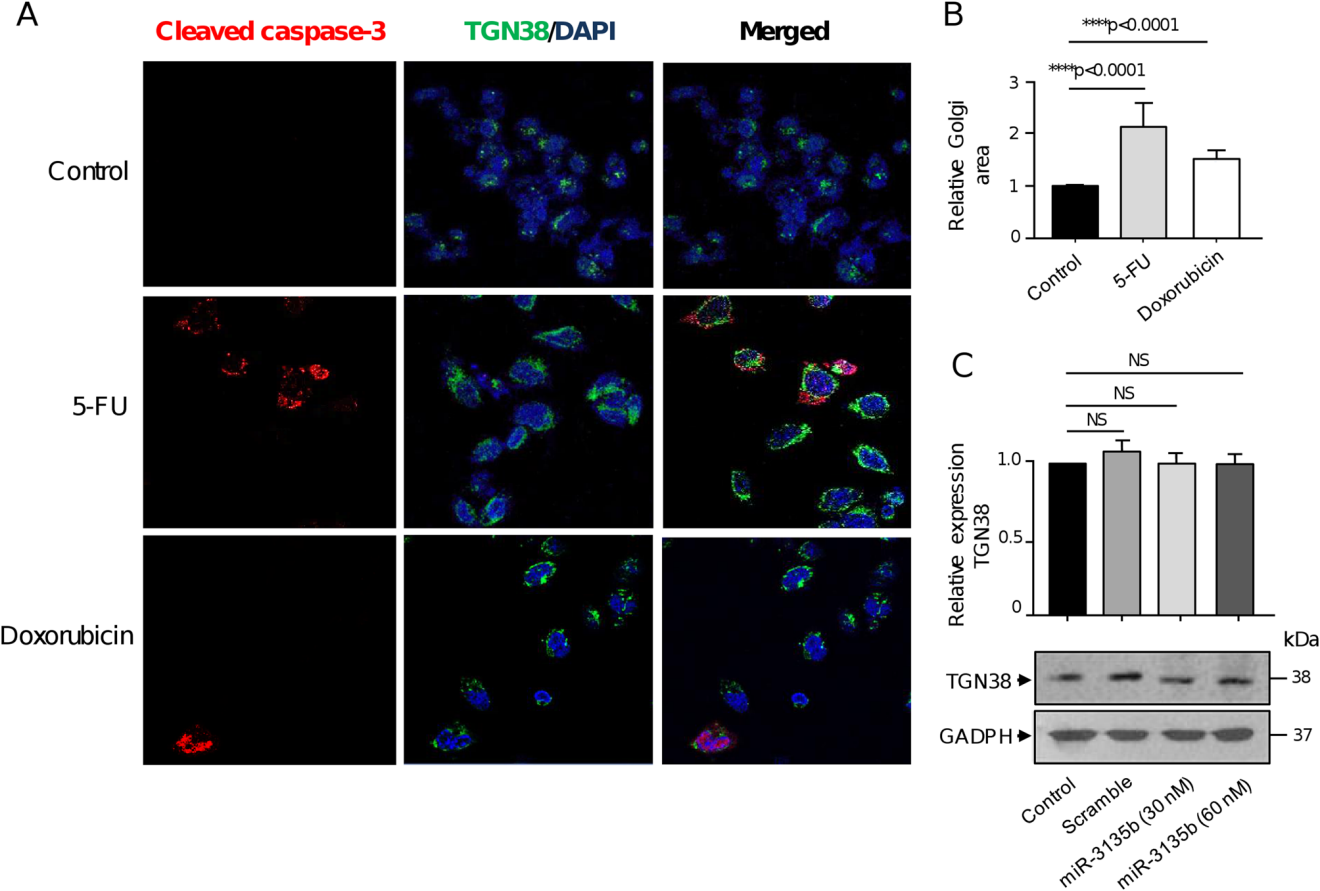
Golgi dispersal is independent of apoptosis. (**A**) Representative immunofluorescence images of TGN38 Golgi protein and cleaved-caspase 3 in control, 5-FU and doxorubicin-treated HCT-15 cells. Samples were incubated with DAPI (blue channel), anti-TGN38 (green channel) and anti-cleaved-caspase-3 (red channel) antibodies. (**B**) Relative quantification of the Golgi area as showed in panel A. (**C**) Cropped images from immunoblots using antibodies against TGN38 in control, scramble, and miR-3135b transfected cells. GADPH was used as endogenous loading control. Complete developed Western blot membranes are provided in Supplementary data 1. Error bars represent the standard deviation of triplicate experiments and *p* values < 0.05 were considered to be statistically significant. NS = non-significant.

To rule out the possibility that Golgi fragmentation could be due to apoptosis activation, we immunodetected the caspase-3 protein in HCT-15 cells using confocal microscopy. As expected, after treatment with 5-FU and doxorubicin, a subpopulation of cells was positive for cleaved caspase-3 representative of apoptotic cells (Fig. 6A, left panel). The staining of cells with TGN38 antibodies also showed that stacks of the Golgi ribbon arrangement appear dispersed in cytoplasm after drugs treatments in comparison with untreated control cells, as previously observed (Fig. 6A, middle panel). In contrast, only a minority of HCT-15 cells showed the double caspase-3/TGN38 staining (Fig. 6A, right panel). Moreover, most non-apoptotic cells exhibited Golgi fragmentation uniformly dispersed throughout the cytoplasm, which was accompanied with an increase in relative Golgi area (Fig. 6A,B). These data strongly suggest that the organelle dispersion was predominantly independent of apoptosis as previously reported in cervical cancer HeLa cells^5^. Next, we also discarded the possibility that expression of TGN38 protein used as trans-Golgi network marker could be targeted by miR-3135b. For this, we performed Western blot assays to detect TGN38 levels in controls and transfected cells with miR-3135b mimics. After densitometric analysis of immunodetected bands, we did not find any significant changes in the expression of the TGN38 protein following the transfection of miR-3135b mimics at 30 and 60 nM concentrations (Fig. 6C).

### MicroRNA-3135b targets the GOLPH3/AKT/mTOR axis

**Figure 7:**
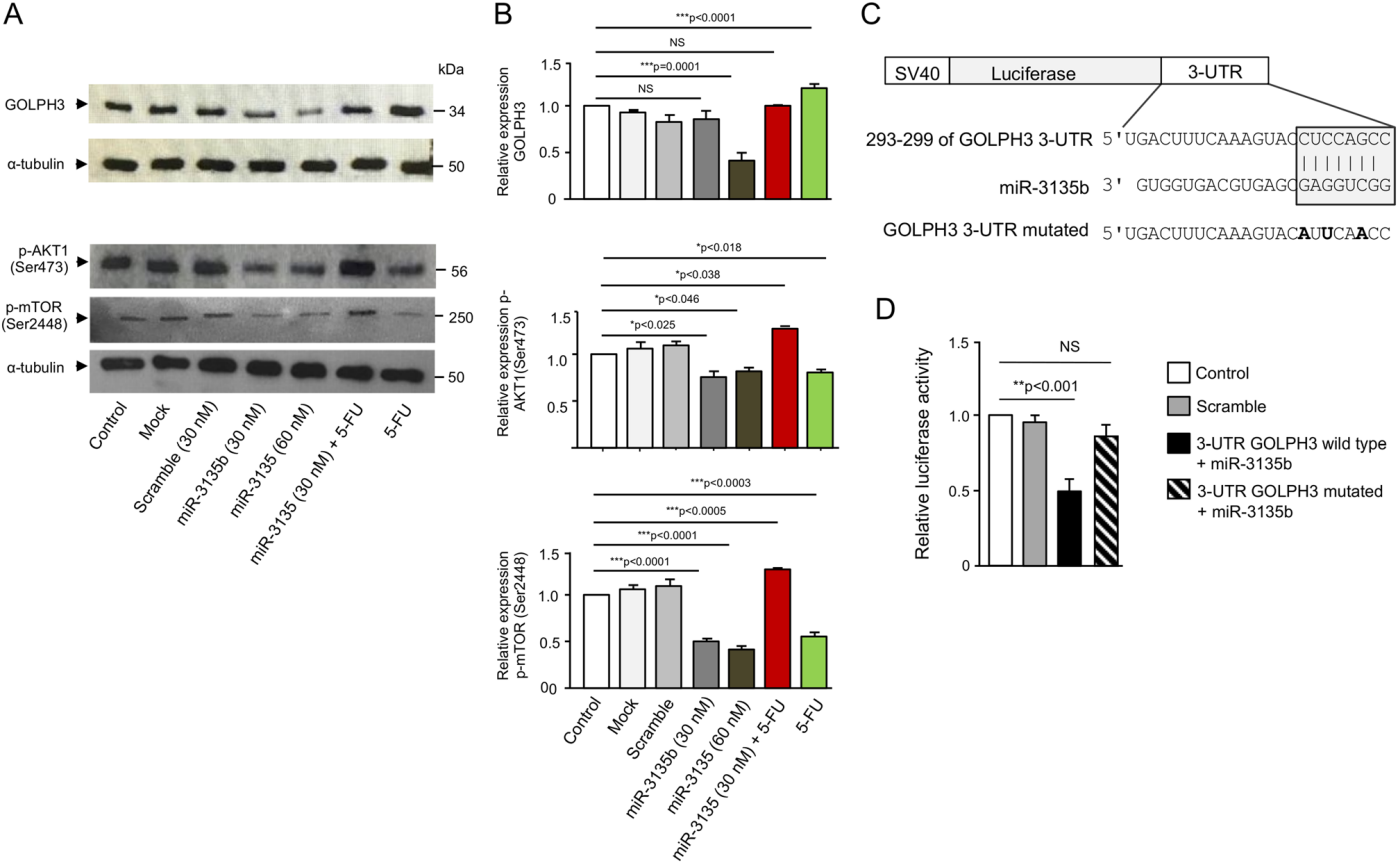
MiR-3135b downregulates GOLPH3 and suppresses the phosphorylation of AKT1 and mTOR in HCT-15 cells. (**A**) Cropped images from immunoblots using antibodies against GOLPH3 (1:500), p-AKT1 (Ser473, 1:1,000) and p-mTOR (Ser2448, 1:1,000) primary antibodies. Anti-α-tubulin antibodies were used as loading control. Complete developed Western blot membranes are provided in Supplementary data 1. (**B**) Densitometric analysis of immunodetected bands in panel A. Data represent the mean ± S.D. of three independent experiments, One-way ANOVA was applied to compare control conditions measurements among treatments while post-hoc Tukey’s comparison was used to obtain *p* values. Images are representative of three independent experiments. (**C**) Schematic representation of p-miR report constructs containing the 3′-UTR of the GOLPH3 cloned downstream of luciferase gene. miR-3135b seed sequence is indicated in gray box. Point mutations in the miR-3135b binding sites of 3′-UTR of the GOLPH3 gene are denoted in bold. (**D**) Luciferase assays in HCT-15 cells transfected with miR-3135b and the constructs described in panel C. Error bars represent the standard deviation of triplicate experiments and *p* values < 0.05 were considered to be statistically significant. NS = non-significant.

To gain insights into the molecular mechanism underlying the protective role of miR-3135b on Golgi dispersal induced by drugs, we investigated the expression levels of GOLPH3, p-AKT1 (Ser473) and p-mTOR (Ser2448) proteins by Western blot assays using non-treated, mock, and scramble transfected HCT-15 cells, as well as cells transfected with miR-3135b mimic alone or in combination with 5-FU. Results showed that the expression of GOLPH3, and p-AKT1 (Ser473) and p-mTOR (Ser2448) proteins does not present significant variations among non-treated, mock and scramble controls (Fig. 7A,B). In contrast, the p-AKT1 (Ser473) and p-mTOR (Ser2448) levels were significantly repressed after miR-3135b mimic transfection at 30 nM and 60 nM, whereas GOLPH3 expression was inhibited only at 60 mM (Fig. 7A,B). Treatment with 5-FU resulted in a significant increase of GOLPH3, and a reduction of p-AKT1 (Ser473) and p-mTOR (Ser2448). No significant changes in α-tubulin expression used as control were found in treated and control cells. In light of these data, it was reliable to propose that miR-3135b may regulate Golgi dispersal caused by chemotherapy through direct binding to its cognate targets. Bioinformatic analysis predicted that 3′-UTR of GOLPH3 gene contains a potential miR-3135b binding site (Fig. 7C). Therefore, to define if miR-3135b can exert a posttranscriptional repression of GOLPH3 gene, we performed luciferase reporter assays. A DNA fragment corresponding to 3′-UTR of GOLPH3 was cloned downstream of the luciferase-coding region of pmiR-LUC vector (Fig. 7C). In addition, a mutated version of the predicted miR-3135b binding site was included as control. Thereafter, wild-type and mutated pmiR-LUC-GOLPH3 and pmiR-LUC control constructs were transfected into HCT-15 cells and luciferase activity was analyzed after 24 h. Data showed that co-transfection of recombinant plasmids and miR-3135b mimics result in a significantly reduction of luciferase activity in comparison to controls (Fig. 7D). In contrast, when the mutated sequence of GOLPH3 3′-UTR was assayed, no significant changes in luciferase activity were observed suggesting that miR-3135b binding was specific.

### Golgi dispersal is associated to autophagy activation

**Figure 8:**
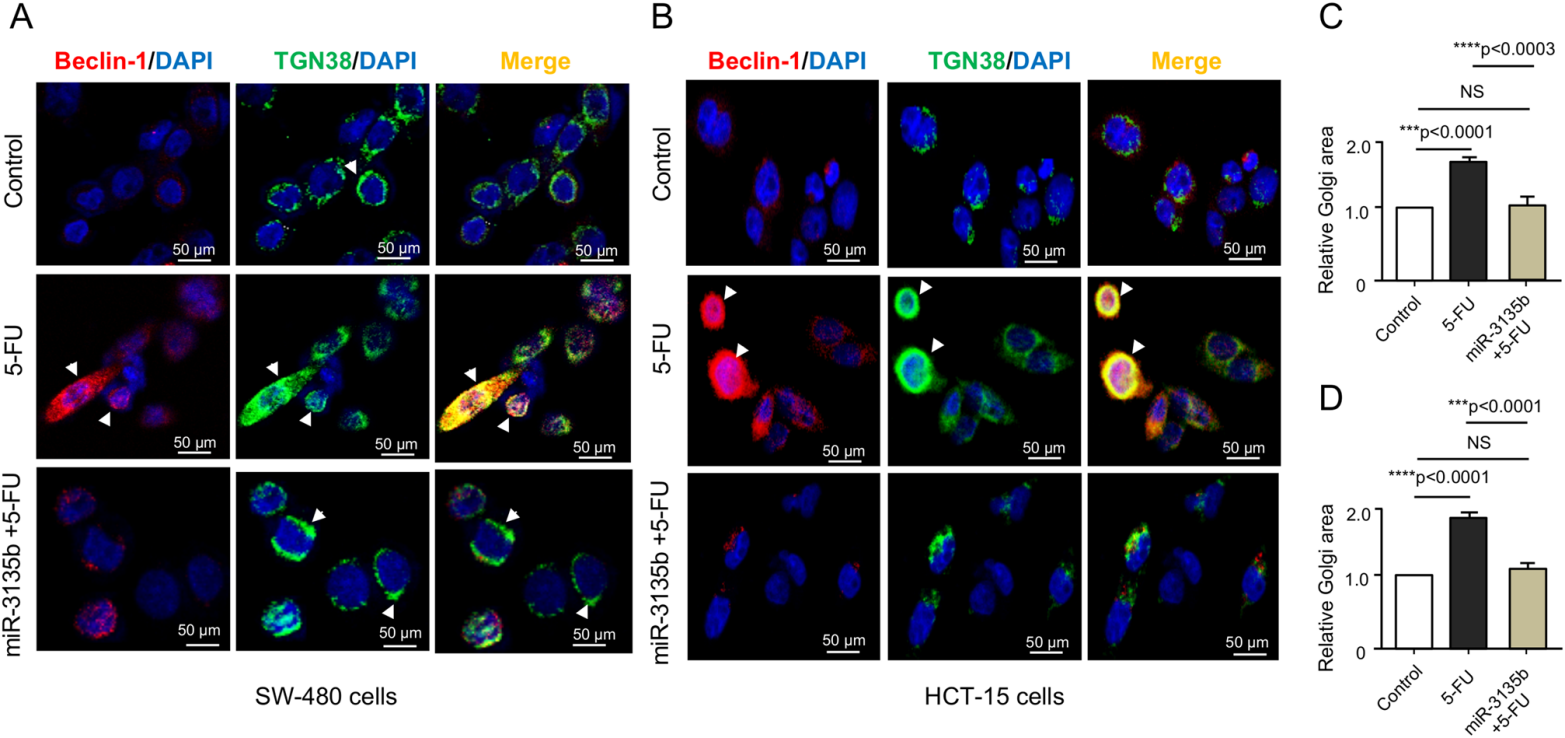
Immunolocalization of beclin-1 and TGN38 in HCT-15 and SW-480 cancer cells. (**A**–**B**) Immunofluorescence assays using antibodies against TGN38 trans-Golgi protein (green channel) and beclin-1 autophagy protein (red channel) in control, 5-FU, and miR-3135b plus 5-FU treated cells. DAPI staining was used to visualize nuclei (blue channel). Relative quantification of the Golgi area of (**C**) SW-480 cells and (**D**) HCT-15 cells. Error bars represent standard deviation from the mean of triplicate and *p* values < 0.05 were considered statistically significant. NS = non-significant.

Recent studies showed that endo-membranous organelles such as endoplasmic reticulum and the Golgi apparatus are essential for the assemblage and production of autophagic-vesicles during the early stages of autophagy activation^10^. In addition, autophagy triggering depends on a functional mTOR pathway. As we observed that dispersal of Golgi in cancer cells treated with 5-FU and doxorubicin was associated with activation of mTOR signaling (and hindered by miR-3135b), we hypothesized that a pro-survival autophagic pathway could be in turn activated in response to chemotherapy, which may result in recycling of Golgi fragments allowing cell survival of cancer cells. To test this hypothesis, we performed immunofluorescence assays using specific antibodies against TGN38 and beclin-1 autophagy protein in HCT-15 and SW-480 cells treated with 5-FU. Data showed that beclin-1 signal is very weak in both non-treated HCT-15 and SW-480 control cells. Remarkably, after treatment with 5-FU, the beclin-1 signal was significantly increased and located around the nucleus in a pattern overlapping with TGN38 and associated to fragmented Golgi, suggesting that ongoing autophagy could be occurring in SW-480 and HCT-15 cells (Fig. 8). The co-immunolocalization of both proteins was better observed in HCT-15 than in SW-480 cells (Fig. 8B). Moreover, the restoration of miR-3135b in 5-FU treated cells led to a significant reduction in Golgi fragmentation and the consequent loss of beclin-1 signal, suggesting the inhibition of autophagy which was accompanied with the recovery of Golgi structure integrity in HCT-15 and SW-480 cells (Fig. 8A–D).

## Discussion

During the last decades, the search for genes and signaling pathways responsible for resistance to chemotherapy in human cancers has been exhaustive. However, it was not expected that a cytoplasmic DNA damage response mechanism operates in response to cytotoxic therapies in cancer cells, changing our assumption that only the nucleus orchestrates the DNA damage response. The effect of DNA damage on Golgi integrity has been largely neglected in past studies as it was thought as a consequence of apoptosis generated by extensive DNA damage. However, a report of Farber-Katz et al., demonstrated that Golgi fragmentation occurs after DNA damage caused by chemotherapeutic drugs as a mechanisms based on the activation of DNA-PK/GOLPH3/MYO18a pathway to allow cancer cells survival, which is largely unrelated to apoptosis^5^. A very limited number of reports have studied the dispersion of Golgi apparatus as a novel survival mechanism involved in the response to DNA damage in cancer cells [^5,16^, this report]. Moreover, the involvement of microRNAs functioning as either oncogenes or tumor suppressors in this phenomenon is largely unknown^25^. Inspired by these facts, here we investigated the functional relationships between miR-3135b and the Golgi apparatus dispersal in response to therapy with DNA-damaging drugs. Our data showed that miR-3135b is downregulated in colorectal cancer cells and clinical tumors suggesting that it could function as tumor suppressor gene (Fig. 1). A previous study using a genome-wide profiling of microRNAs evidenced that miR-3135b is repressed in stool samples of patients with colorectal adenocarcinoma^26^, but no functional analysis of this particular microRNA was performed. On the other hand, several studies indicated that microRNAs might regulate GOLPH3 expression^27–30^. Here, we found that overexpression of miR-3135b inhibits cell proliferation, and also has a synergistic effect with 5-FU treatment in activating apoptosis, perhaps through repression of GOLPH3, which is consistent with previous studies that have shown that the inhibition of GOLPH3 by miR-126 promotes apoptosis and inhibits cell proliferation in esophageal squamous cell carcinoma^27^. Likewise, miR-134 suppressed cell proliferation by direct binding to the 3′-UTR of GOLPH3 in gastric cancer cells^28^. Cell cycle assays showed that miR-3135b+5-FU treatment increases the percentage of cells in G1 phase and decreases the ratio of S phase cells in both cell lines in comparison with control cells. Here, we also evaluated the functional relationships between miR-3135b and AKT1/mTOR signaling which is active in almost all cancers. These pathways have been largely associated to cell survival, resistance to chemotherapy and metastasis in diverse types of malignancies^31,32^. For instance, it was reported that miR-10a significantly inhibits cell proliferation and migration, and promotes apoptosis of MCF-7 breast cancer cells, which was attributed to suppression of phosphorylation levels of AKT and mTOR^33^. Our data showed that overexpression of miR-3135b in HCT-15 cells results in reduction of p-AKT1 (Ser473) and p-mTOR (Ser2448) levels, which may explain, in part, the inhibition of cell proliferation. Interestingly, and consistent with AKT1/mTOR pathways activation, other reports showed that the dispersion of the Golgi apparatus via GOLPH3 has the ability to enhance the hypersialylation of RTK receptors by promoting the transduction of the PI3K/AKT/mTOR signaling^34,35^.

On the other hand, it was shown that damage to DNA with doxorubicin and camptothecin triggers the dispersion of the Golgi through the cytoplasm and activates the protein kinase of DNA damage and DNA-PK GOLPH3/MYO18A axis^5^. We also found that exposure to 5-FU induces fragmentation of Golgi apparatus, which was hindered by miR-3135b in HCT-15, and SW-480 colorectal cancer cells. Thus, by simulating a cellular environment of DNA damage using 5-FU, we found novel findings on the regulation of Golgi dispersal by miR-3135b, maybe by targeting GOLPH3, adding a piece to the puzzle. These findings are in agreement with data showing that inhibition of GOLPH3 with an interfering RNA also prevents dispersion of the Golgi apparatus in response to doxorubicin^5,30,36^.

Interestingly, we found a potential functional link between Golgi dispersal, autophagy activation and miR3135b. It is well known that endoplasmic reticulum and the Golgi apparatus are pivotal for the assemblage of autophagic-vesicles^10^. Our data showed that the autophagy protein beclin-1 is located at vesicles associated with the fragmented Golgi, suggesting that ongoing autophagy could be occurring in response to drug treatment to allow cell survival (Fig. 8). We found that cells with excessive Golgi dispersion exhibit strong staining for beclin-1; conversely, cells that maintained the structure of the Golgi apparatus presented a low beclin-1 signal, which could indicate that the activation of autophagy has a direct correlation with the loss of the Golgi structure and mTOR activation in cancer cells. A similar mechanism on the dependence of Golgi ribbon structure, and autophagy activation via mTOR has also been documented in previous studies^37^.

**Figure 9:**
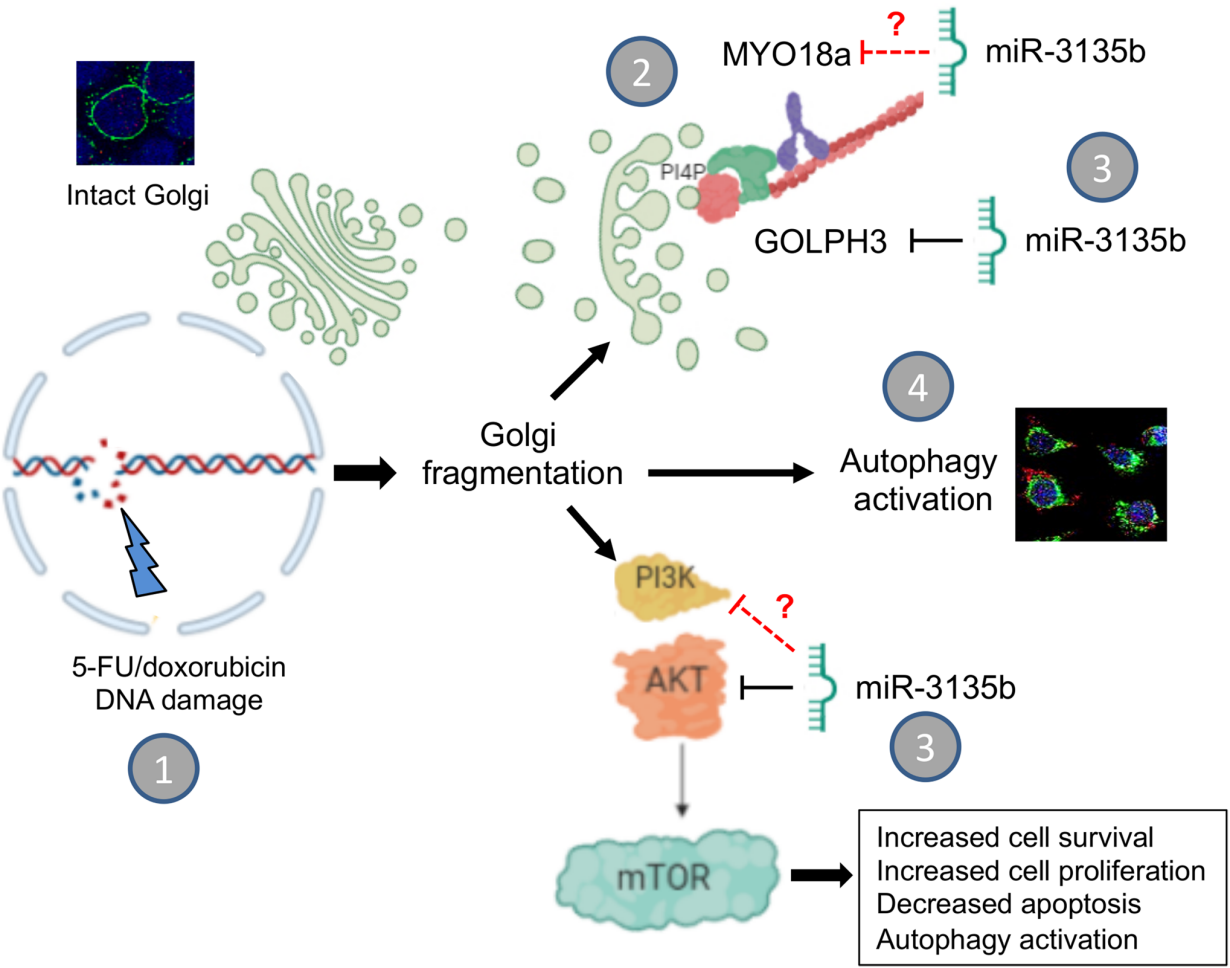
Working model of the role of miR-3135b in the prevention of Golgi dispersal induced by chemotherapy agents. (**1**) DNA damage caused to cancer cells by 5-FU and doxorubicin drugs promotes the dispersion of Golgi apparatus. (**2**) Part of the previously described mechanism established that after DNA damage, GOLPH3 increases its affinity for myosin MYO18b resulting in tensile forces with F-actin cytoskeleton inducing Golgi fragmentation to confers cell survival and resistance to DNA-damaging agents^5,6^. (**3**) Here, we showed that ectopic overexpression of miR-3135b inhibits the Golgi dispersal, cell survival, cell cycle and sensitizes cells to 5-FU treatment, maybe by targeting GOLPH3 and AKT1/mTOR signaling. (**4**) In addition, 5-FU activates the autophagy associated to Golgi fragmentation which is hindered by miR-3135b mimics. Non-confirmed miR-3135b potential targets participating in the mechanism are indicated by discontinuous red lines.

We are aware that the present report has limitations that should be addressed in future works: (1) this is a basic research study which must be further confirmed by the analysis of miR-3135b and GOLPH3/AKT1/mTOR axis molecules in biopsies from CRC patients in order to evaluate their clinical value; (2) it is necessary to assess the protective role of miR-3135b in Golgi dispersal in response to other drugs and radiotherapy in a larger number of cell lines from diverse types of cancer; and (3) to characterize additional miR-3135b gene targets involved in the cytoplasmic DNA damage response. In conclusion, our results demonstrate that the GOLPH3/AKT/mTOR axis is negatively regulated by miR-3135b resulting in apoptosis activation and a decreased proliferative capacity and cell cycle arresting of HCT-15 and SW-480 cancer cells (Fig. 9). We also hypothesize that a pro-survival autophagic pathway could be activated which may result in recycling of Golgi fragments allowing cell survival of CRC cells in response to drugs treatments (Fig. 9). Our data also provide new insights into the role of microRNAs in the mechanisms of cytoplasmic DNA damage response, and suggest that manipulation of miR-3135b levels could be a potential therapeutic tool in colorectal cancer.

## Methods

### Cell lines

Human colorectal cancer cell lines HCT-15 (ATCC, CCL-225), SW-480 (ATCC, CCL-228), and Caco2 (ATCC, HTB-37), were obtained from the American Type Culture Collection (ATCC), and routinely grown in Dulbecco's modification of Eagle's minimal medium (DMEM) supplemented with 10% fetal bovine serum and penicillin–streptomycin (50 unit/ml; Invitrogen, Carlsbad, CA, USA) in a 5% CO_2_ atmosphere at 37 °C.

### Bioinformatics analysis

The prediction of genes potentially regulated by miR-3135b in the AKT-mTOR pathways was performed using Targetscan^38^ (https://www.targetscan.org/vert_72/) and miRmap^39^ (https://mirmap.ezlab.org/) softwares.

### Validation of miR-3135b expression in an independent cohort

Clinical and microRNAs microarray expression data from 1513 colorectal cancer patients were obtained from the Gene Expression Omnibus accession GSE115513 (PMID: 26740022) previously reported^24^. The differential expression analysis was carried out with the limma package (PMID: 25605792) using R environment, considering FDR < 0.05 as statistically significant.

### RNA isolation

Total RNA was extracted from HCT-15, SW-480, Caco2 and CRL-1790 cells using TRIzol^®^ (Invitrogen) according to the manufacturer’s instructions. RNA integrity was assessed by electrophoresis in 1% TAE agarose gel and bands were visualized with GelRed^®^ staining.

### Reverse transcription and real-time polymerase chain reaction (RT-PCR)

cDNA was synthesized from total RNA using TaqMan MicroRNA Assay (Applied Biosystems, Foster City, CA). 50 ng of total RNA were used for RT assays using a specific miR-3135b stem loop primer, dNTP (100 mM), MultiScribe reverse transcriptase (50 U/µl), 10X buffer, RNase inhibitor (20 U/µl) and RNase-free water. These reactions were incubated in the thermal cycler at 16 °C for 30 min, 42 °C for 5 min and 85 °C for 5 min. Real-time PCR was carried out in a GeneAmp System 9,700 (Applied Biosystems) using RT product, TaqMan Universal PCR master mix, RNase-free water and miR-3135b probes from the TaqMan MicroRNA Assay protocol kit. Reactions were incubated in a 96-well plate at 95 °C for 10 min, followed by 40 cycles at 95 °C for 15 s and 60 °C for 1 min. RNU44 small RNA was used as control for normalizing miRNA data.

### Transfection of microRNA-3135b mimic

miR-3135b mimic (PM20623) and pre-miR-negative (Thermo Fisher Scientific, Inc), were transfected into HCT-15 and SW480 cells using Lipofectamine 2000 transfection reagent (Invitrogen) in Opti-MEM Reduced Serum Medium (Life Technologies). miR-3135b and pre-miR-negative Scramble at 30 nM and 60 nM were added to 6-well plate containing 2 × 10^5^ cells cultured in DMEM for 48 h. To analyze the effectiveness of transfection, a quantitative RT-PCR assay was performed to measure the levels of miR-3135b at 48 h post-transfection.

### Cell proliferation assay

5 mg/ml of MTT reagent ([3-[4,5-dimethylthiazol-2-yl]-2,5 diphenyltetrazolium bromide]) was added for 4 h in the HCT-15 cultures at 37 °C. To solubilize the dye, cells were then mixed with 100 µl of acidified isopropanol (0.04 M HCl) for 15–30 min with gentle shaking. Then, the absorbance in each well was measured at 570 nm in a microplate spectrophotometer. Data were analyzed using the Graph-Pad prism 6 software. For 5-FU and doxorubicin studies, HCT-15 cells (1 × 104) transfected with miR-3135b mimics (30 nM) or scramble (30 nM), were treated with 5-FU (45 μM) for 12 h.

### Apoptosis assays

Death of HCT-15 cells was quantified by Annexin-V and Propidium iodide (PI) reagents from Annexin-V-FLUOS Staining Kit (Roche). For assays, HCT-15 cells (1 × 10^6^) transfected with miR-3135b precursor or treated with the indicated doses of 5-FU or doxorubicin, were resuspended in 100 µl of Hepes buffer with 2 µl of Annexin V and 2 µl of propidium iodide according to the manufacturer's recommendations. Cells were analyzed in a FACSCALIBUR flow cytometer (BD Biosciences).

### Cell cycle assays

Cell cycle assays were made by cycleTEST plus DNA reagent kit (BD Biosciences). Cells were previously washed with 1X PBS and the concentration was adjusted to 1 × 10^6^ cells/ml with Buffer Solution (sodium citrate buffer). For the staining procedure, cells were successively incubated in solutions A, B, and C for 10 min each at room temperature. Then, samples were examined in the flow cytometer FACS Aria and data were analyzed with Modfit LT software (Verity Software House, Inc.)

### Western blot assays

Proteins were separated by electrophoresis in 12% and 6% polyacrylamide gels according to molecular weight. Then, they were transferred to 0.2 μm nitrocellulose membranes (Bio-Rad) using transfer buffer (25 mM Tris, 190 mM glycine and 20% methanol). Membranes were blocked for 60 min at 37 °C with 3% bovine serum albumin (BSA) in TBST-1X (150 mM NaCl, 20 mM Tris, 0.1% Tween-20, at pH 7.5) and incubated overnight at 4 °C with mouse anti-tubulin (1:2,000 Cell Signaling), mouse anti-GOLPH3 (1:1,000 Santa Cruz), rabbit anti-H2A.X (1:500, Cell Signaling), mouse anti-TGN38 (1:500 Santa Cruz), rabbit anti-phospho AKT1 (1:1,000, Cell Signaling), and rabbit anti-phospho mTOR (1:1,000 Cell Signaling) antibodies. After washing, membranes were incubated with anti-rabbit or anti-mouse secondary antibodies (1:2,500) (Jackson ImmunoResearch). Chemiluminiscent detection of immunodetected bands was performed using the ECL Western blot detection reagent (Amersham).

### Immunofluorescence assays and confocal microscopy

HCT-15 and SW-480 cells transfected with miR-3135b precursor or treated with the indicated doses of 5-FU and doxorubicin for 12 h, followed by 24 h of recovery, were seeded on coverslips (1 × 10^3^ cells/cm^2^). After 48 h, cells were washed three times with 1 × PBS buffer and fixed with 4% paraformaldehyde buffer for 30 min at 37 °C. Then, cells were permeabilized with Triton-X 100 for 5 min, blocked with 1% of PBS-BSA, and incubated with anti-TGN38 (1:500, Santa Cruz), anti-cleaved caspase 3 (1:1,000, Cell Signaling) or anti-beclin-1 (1:50, Cell Signaling) antibodies. Finally, slides were assembled with vectashield/DAPI solution and documented using a confocal microscope. For measurement of Golgi area, images of HCT-15 and SW-480 cells stained with Golgi marker TGN38 antibodies were quantified by demarcation of the Golgi area with the polygon tool and calculation of its area using the ImageJ program.

### Transmission electronic microscopy

HCT-15 cells were fixed with 2% glutaraldehyde in 0.1 M Cacodylate buffer at pH 7.4 for 1 h at room temperature. Subsequently, they were treated with 1% Osmium Tetroxide (OsO4) in Cacodylate for 1 h in the dark at 4 °C and dehydrated in increasing concentrations of ethanol. Then, samples were embedded in Polybed epoxy resins and polymerized at 60 °C for 24 h. Thin sections (60 nM) were obtained and stained with uranyl acetate and lead citrate for examination under a Philips Morgagni 268 electron microscope.

### Luciferase reporter gene assays

The 3′-UTR sequence (wild-type and mutant) of the GOLPH3 gene was cloned downstream of the luciferase gene in the p-miR-report vector (Ambion) and recombinant plasmids were verified through automatic sequencing. The recombinant wild type and mutant pmiR-GOLPH3 plasmids and the p-miR-report control plasmid were transfected into HCT-15 cells using lipofectamine 2000 (Invitrogen). Then, cells were mixed with 1 × lysis buffer and transfered to a new tube. Finally, 20 µl of cell lysate were mixed with 100 µl of luciferase assay reagent and the light produced was measured with a Fluoroskan Ascent™ Microplate Fluorometer (Thermo Scientific).

### Statistical analysis

To compare means of more than two variables, one-way analysis of variance (ANOVA) followed by post-hoc test (Tukey) were used and *p* value lower than 0.05 was considered statistically significant. Experiments were performed three times by triplicate and results were represented as mean ± S.D. For miR-3135b expression assays in cells treated with drugs, we performed Student's *t* test to compare the expression of miR-3135 between two variables and *p* < 0.05 was considered statistically significant. Experiments were performed three times by triplicate and results were represented as mean ± S.D. The miR-3135b expression analysis in the previously published data sets from microarrays miRNAs profiling of 1893 normal/colorectal carcinoma-paired samples, and 290 adenoma tissues samples deposited in NCBI GEO database (GSE115513)^24^, was carried out using the Limma package in R (www.bioconductor.org/packages/release/bioc/html/limma.html) employing gene-wise linear models and empirical Bayes methods. We have taken in account the multiple testing correction by considering an FDR < 0.05 as statistically significant.

## Supplementary information

Supplementary information
