# Supplementary material for: A novel protective role for microRNA-3135b in Golgi apparatus fragmentation induced by chemotherapy via GOLPH3/AKT1/mTOR axis in colorectal cancer cells
Source: Sci Rep. 2020 Jun 29;10:10555. doi: 10.1038/s41598-020-67550-0 (PMC7324564; doi:10.1038/s41598-020-67550-0)
Supplement: Supplementary file 1 — Supplementary information [file 41598_2020_67550_MOESM1_ESM.pdf]

A novel protective role of microRNA-3135b in Golgi apparatus fragmentation induced by chemotherapy via GOLPH3/AKT1/mTOR axis in colorectal cancer cells

Stephanie I. Núñez-Olvera[1], Bibiana Chávez-Munguía[2], María Cruz del Rocío Terrones-Gurrola[3], Laurence A. Marchat[4], Jonathan Puente-Rivera[5], Erika Ruíz-García[6], Alma D. Campos-Parra[7], Carlos Vázquez-Calzada[2], Erik R. Lizárraga-Verdugo[8], Rosalío Ramos-Payán[8], Yarely M. Salinas-Vera[1], César López-Camarillo[1]*

[1]Posgrado en Ciencias Genómicas, Universidad Autónoma de la Ciudad de México, CDMX, México. [2]Departamento de Infectómica y Patogénesis Molecular, CINVESTAV-IPN, CDMX, México. [3]Coordinación Académica Región Altiplano, Universidad Autónoma de San Luis Potosí, San Luis Potosí, México. [4]Programa en Biomedicina Molecular y Red de Biotecnología, Instituto Politécnico Nacional, CDMX, México. [5]Departamento de Ecología Funcional, Instituto de Ecología, Universidad Nacional Autónoma de México, CDMX, México. [6]Laboratorio de Medicina Translacional y Departamento de Tumores Gastro-Intestinales, Instituto Nacional de Cancerología, CDMX, México. [7]Laboratorio de Genómica, Instituto Nacional de Cancerología, CDMX, México. [8]Facultad de Ciencias Químicas Biológicas, Universidad Autónoma de Sinaloa, Sinaloa, México.

*****Corresponding author: Dr. César López-Camarillo. Universidad Autónoma de la Ciudad de México. San Lorenzo 290 col del Valle. C.P. 03100. CDMX, México. Email address: cesar.lopez@uacm.edu.mx ORCID: 0000-0002-9417-2609.

Supplementary Figure 1. Complete membranes for Western blot assays described in the main text and figures. The name of dececeted proteins is indicated in each membrane.

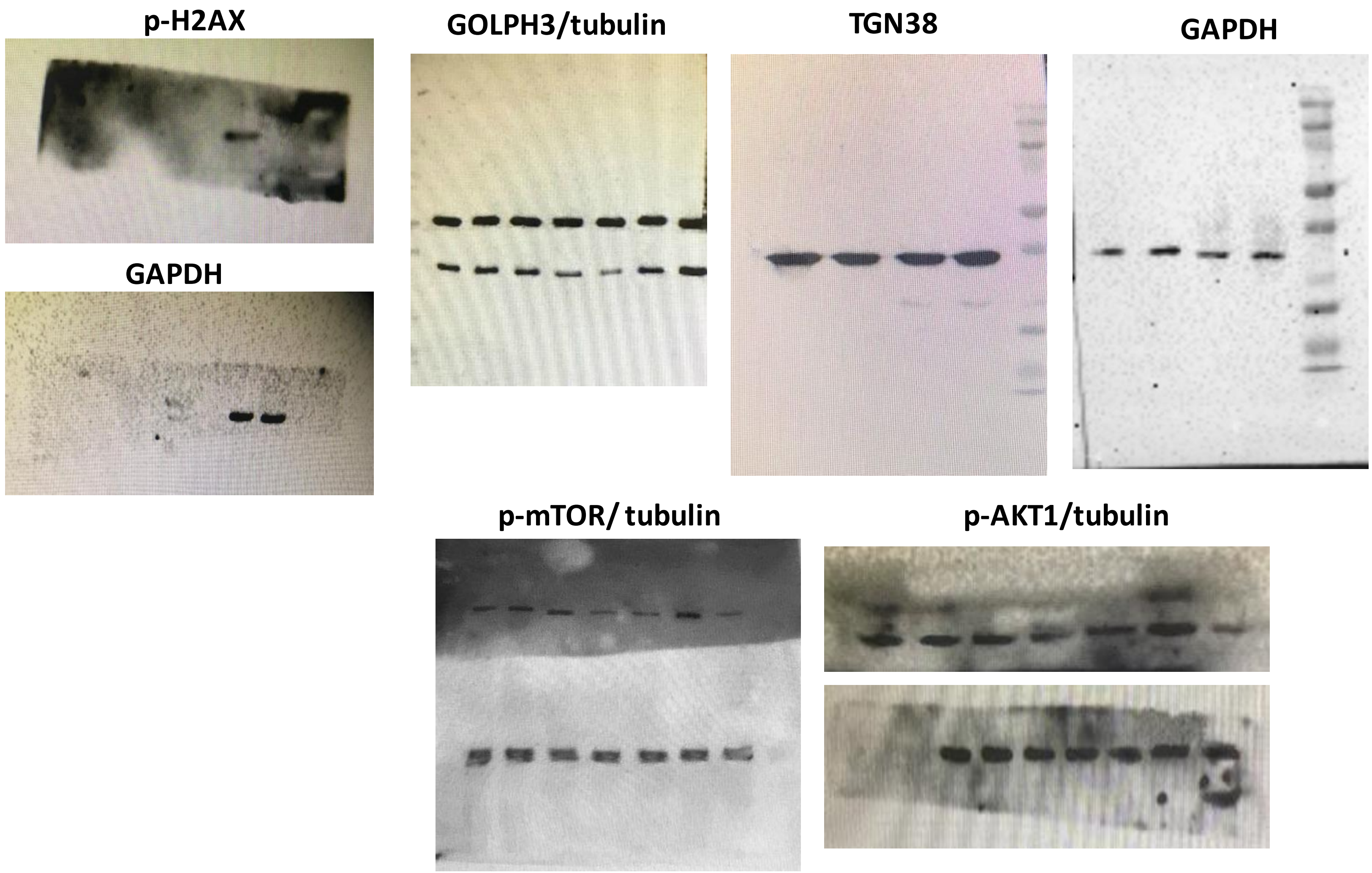

miR-3135b mimics transfection in CRC cells

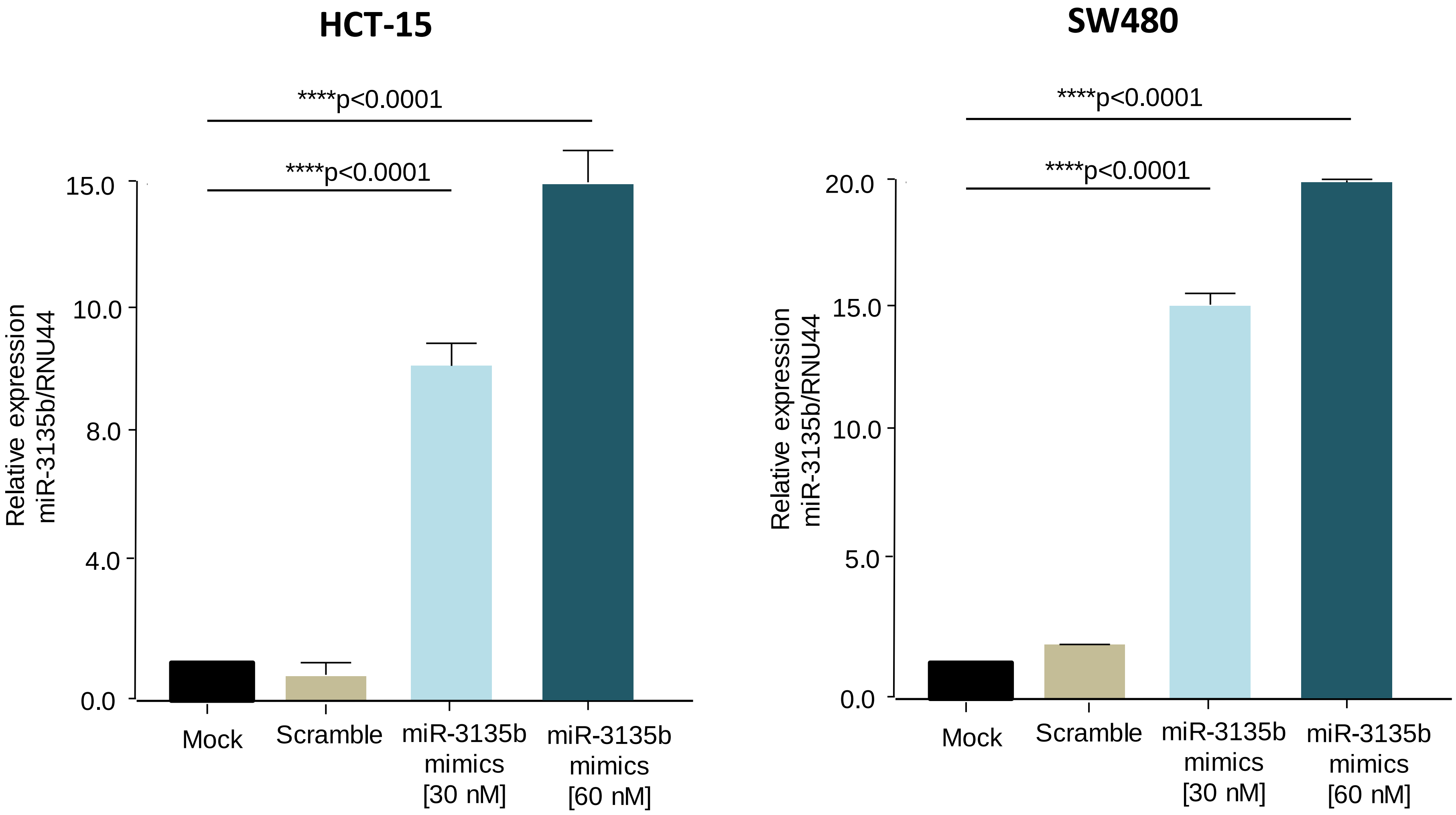

Supplementary figure 2. Validation of miR-3135b overexpression in colorectal cancer cells transfected with RNA mimics. Bars show expression of miR-3135b after transfection with mimic precursor at concentration of 30 and 60 nM compared to mock and scramble conditions. The error bars represent the standard error of the mean of three experiments in triplicate, the value of $p < 0.05$ was considered significant.
